# Supplementary material for: Environmental DNA Metabarcoding Reveals Divergent Patterns of Biodiversity, Community Assembly, and Environmental Sensitivity Across Taxa in Adjacent Rivers
Source: Biology (Basel). 2025 Dec 17;14(12):1796. doi: 10.3390/biology14121796 (PMC12730359; doi:10.3390/biology14121796)
Supplement: Supplementary file 1 [file biology-14-01796-s001.zip › Table_S1.pdf]

Table S1. Land use composition in the riparian zones of the Tuhai River and Chao River

Land Use Type	Chao River (km^2)	Percentage (%)	Tuhai River (km^2)	Percentage (%)
Cropland	516214.8	67.47%	1541110.5	74.87%
Grassland	0.9	0.00012%	82.8	0.004%
Water	83608.2	10.93%	126521.1	6.15%
Barren	6313.5	0.83%	6773.4	0.33%
Impervious	158985.0	20.78%	383820.3	18.65%
