# Supplementary material for: Environmental DNA Metabarcoding Reveals Divergent Patterns of Biodiversity, Community Assembly, and Environmental Sensitivity Across Taxa in Adjacent Rivers
Source: Biology (Basel). 2025 Dec 17;14(12):1796. doi: 10.3390/biology14121796 (PMC12730359; doi:10.3390/biology14121796)
Supplement: Supplementary file 1 [file biology-14-01796-s001.zip › Table_S2.pdf]

Table S2. Water quality parameters in two adjacent rivers: a comparative analysis.

	Chao River	Tuhai River
EC（μs/cm）	8.18 ± 5.07	20.24 ± 16.81
WT（℃）	22.51 ± 0.89	22.38 ± 2.40
DO（mg/L）	11.40 ± 4.11	9.63 ± 3.45
pH	8.43 ± 0.55	8.45 ± 0.42
TP（mg/L）	0.09 ± 0.07	0.02 ± 0.01
TN（mg/L）	2.59 ± 1.28	1.60 ± 0.95
NH_4-N（mg/L）	1.07 ± 0.43	0.77 ± 0.26
COD（mg/L）	56.67 ± 36.77	86.87 ± 83.84
BOD_5（mg/L）	17.55 ± 12.80	26.75 ± 29.89
