# Supplementary material for: Environmental DNA Metabarcoding Reveals Divergent Patterns of Biodiversity, Community Assembly, and Environmental Sensitivity Across Taxa in Adjacent Rivers
Source: Biology (Basel). 2025 Dec 17;14(12):1796. doi: 10.3390/biology14121796 (PMC12730359; doi:10.3390/biology14121796)
Supplement: Supplementary file 1 [file biology-14-01796-s001.zip › Table_S3.pdf]

Table S3.Water Quality Parameters and Their Measurement Methods

Water Quality Parameter	Measurement Method
Total Nitrogen (TN)	Alkaline potassium persulfate digestion followed by ultraviolet spectrophotometry
Ammonium Nitrogen (NH_4-N)	Quantified using Nessler's reagent spectrophotometry
Total Phosphorus (TP)	Measured via ammonium molybdate spectrophotometry
Biochemical Oxygen Demand (BOD_5)	Assessed using the dilution and inoculation method
Chemical Oxygen Demand (COD)	Analyzed by acid potassium permanganate titration
